# StMYB44 negatively regulates phosphate transport by suppressing expression of *PHOSPHATE1* in potato

**DOI:** 10.1093/jxb/erx026

**Published:** 2017-03-01

**Authors:** Xiangjun Zhou, Manrong Zha, Jing Huang, Li Li, Muhammad Imran, Cankui Zhang

**Affiliations:** 1 Department of Agronomy, Purdue University, West Lafayette IN 47907, USA; 2 Robert W. Holley Center for Agriculture and Health, USDA-ARS, Cornell University, Ithaca, NY 14853, USA; 3 Department of Soil and Environmental Sciences, University College of Agriculture, University of Sargodha, Pakistan 40100

**Keywords:** Gene expression, phosphate deficiency, PHOSPHATE1, potato, protein–protein interaction, RNA-Seq, StMYB44

## Abstract

Phosphorus is an important macronutrient for plant growth, but often deficient in soil. To understand the molecular basis of the complex responses of potato (*Solanum tuberosum* L.) to phosphate (Pi) deficiency stress, the RNA-Seq approach was taken to identify genes responding to Pi starvation in potato roots. A total of 359 differentially expressed genes were identified, among which the *Solanum tuberosum* transcription factor gene *MYB44* (*StMYB44*) was found to be down-regulated by Pi starvation. *StMYB44* was ubiquitously expressed in potato tissues and organs, and StMYB44 protein was exclusively localized in the nucleus. Overexpression of *StMYB44* in potato resulted in lower accumulation of Pi in shoots. Transcriptomic analysis indicated that the abundance of *S. tuberosum PHOSPHATE1* (*StPHO1*), a Pi transport-related gene, was reduced in *StMYB44* overexpression lines. In contrast, knock-out of *StMYB44* by a CRISPR/Cas9 system failed to increase transcription of *StPHO1*. Moreover, StMYB44 was found to interact in the nucleus with AtWRKY6, a known Arabidopsis transcription factor directly regulating *PHO1* expression, and StWRKY6, indicating that StMYB44 could be a member of the regulatory complex controlling transcription of *StPHO1*. Taken together, our study demonstrates that *StMYB44* negatively regulates Pi transport in potato by suppressing *StPHO1* expression.

## Highlight

Pi deficiency suppressed the transcription factor gene *StMYB44*, encoding the protein that interacts with Arabidopsis WRKY6 and StWRKY6 and negatively affects Pi distribution by suppressing the expression of *PHOSPHATE1* in potato.

## Introduction

Inorganic phosphates (Pi) are taken up by plants to meet the phosphorus (P) requirements for a variety of structural and physiological functions. Inadequate supply of P in soil negatively affects plant development and growth (Raghothama, 1999). Reshaping root architecture and development is one of the mechanisms to increase P_i_ uptake, mobilization, and utilization upon P_i_ deficiency (Devaiah *et al.*, 2007*b*). Changing expression of Pi-responsive genes, and altering metabolic and developmental processes are molecular adaptations in this regard (Wu *et al.*, 2003; Thibaud *et al.*, 2010; Secco *et al.*, 2013; Puga *et al.*, 2014). Systematic transcriptional regulation of Pi-responsive genes is believed to be the major regulatory step in maintaining Pi homeostasis (Hammond *et al.*, 2004). A number of transcription factors mediating plant responses to Pi starvation have been identified in Arabidopsis and rice, including MYB transcription factors, PHR1/OsPHR2, PSR1, AtMYB2, MYB62, and OsMYB2P-1 (Wykoff *et al.*, 1999; Rubio *et al.*, 2001; Zhou *et al.*, 2008; Devaiah *et al.*, 2009; Dai *et al.*, 2012; Baek *et al.*, 2013), WRKY transcription factors AtWRKY6, AtWRKY42, AtWRKY45, and AtWRKY75 (Devaiah *et al.*, 2007*a*; Chen *et al.*, 2009; H. Wang *et al.*, 2014; Su *et al.*, 2015), basic helix–loop–helix transcription factors OsPTF1 and bHLH32 (Yi *et al.*, 2005; Chen *et al.*, 2007, zinc-finger transcription factor ZAT6 (Devaiah *et al.*, 2007*b*), and APETALA2/ETHYLENE RESPONSE FACTOR, AtREF070 (Ramaiah *et al.*, 2014). Each transcription factor specifically activates or suppresses a single or multiple Pi-related genes in response to Pi starvation (Chen *et al.*, 2009; H. Wang *et al.*, 2014; Su *et al.*, 2015). Nuclear proteins SPX1 and SPX2 carry an SPX domain, which exists in Pi sensors and other Pi starvation signaling proteins in yeast and plants. These proteins are found to inhibit the activity of PHR1 and OsPHR2 transcription factors by protein–protein interactions in response to Pi availability in Arabidopsis and rice (Puga *et al.*, 2014; Z. Wang *et al.*, 2014). It demonstrates the vital role of transcription factors in Pi signaling pathways by linking Pi perception and gene expression. Hence, identification of additional transcription factors will further broaden our understanding about the signaling process in plant responses to Pi deficiency.

Among various MYB families, R2R3-type transcription factors are the largest MYB family in plants (Stracke *et al.*, 2001). Based on amino acid sequence similarities, 126 Arabidopsis R2R3-type MYB transcription factors are categorized into 22 subgroups, and the last subgroup of MYB transcription factors mainly mediates hormone signaling and abiotic stress responses (Jung *et al.*, 2008). One of its members, AtMYB77, mediates auxin signaling by interacting with auxin response factors and regulating expression of auxin-inducible genes to control lateral root growth and development (Shin *et al.*, 2007). Another member of this subgroup, AtMYB44, positively regulates drought tolerance by enhancing stomatal closure (Jung *et al.*, 2008). In addition, AtMYB44 has also been shown to induce expression of *ETHYLENE INSENSITIVE2* (*EIN2*), a central component in the ethylene signaling pathway (Liu *et al.*, 2011). Interaction of MYBR1/AtMYB44 with ABA receptor PYR1-LIKE8 (PYL8) mediates leaf senescence and responds to stress and wounding (Jaradat *et al.*, 2013), implying that members of this subgroup are involved in diverse physiological processes in plants.

Potato (*Solanum tuberosum* L.), the fourth largest food crop in the world, faces an array of abiotic stresses including drought, cold, and mineral deficiency (Leone *et al.*, 1999). Unlike Arabidopsis and rice, little is known about the mechanisms to maintain mineral homeostasis in potato since relatively few genes involved in regulation of mineral uptake and distribution have been identified in this species.

The present study was designed to carry out RNA-Seq-based identification of genes, particularly those encoding transcription factors, whose expression is affected in potato roots by Pi starvation. The current study is to explore how StMYB44 (previously named tuber-specific and sucrose-inducible element-binding factor), one of the transcription factors identified, is involved in regulation of Pi uptake and distribution in potato plant.

## Materials and methods

### Plant materials and growth conditions

Tetraploid potato (*Solanum tuberosum* L.), Désirée, plants were grown in a greenhouse under a 14 h light/10 h dark regime at 25 °C. *Arabidopsis thaliana* (ecotype Columbia) were grown in a growth chamber under a 14 h light/10 h dark cycle at 23 °C. Hoagland solution was used in hydroponic growth of potato plants, and was changed every other day. The Pi starvation was initiated by withdrawing Pi from the Hoagland solution when the potato plants were 1 month old. Roots were collected 5 d after the treatment and stored at –80 °C before RNA extraction.

### Plasmid construction, and transformation of Arabidopsis and potato

The coding region of *StMYB44* without the stop codon was amplified by PCR and cloned into the pAVA393 vector (von Arnim *et al.*, 1998) to make the *StMYB44:GFP* fusion gene, which was then subcloned behind a double *Cauliflower mosaic virus* (CaMV) 35S promoter in the binary vector pCAMBIA1300S (Zhou *et al.*, 2015). The complete vector was verified by sequencing and transformed into *Agrobacterium tumefaciens* GV3101 by electroporation. Arabidopsis transformation was performed by the floral-dip method (Clough and Bent, 1998).

For the CRISPR/Cas9 (clustered regularly interspaced short palindromic repeat/Cas9) vector, the sequence GAAGATGATACTATCATCAGG of the *StMYB44* gene was used as the target sequence. Two primers were synthesized and annealed to form the dsDNA and cloned between two *Bsa*I sites of the pKSE401 vector by Golden Gate cloning (Xing *et al.*, 2014). The complete vector was verified by sequencing.

The 1.5 kb *StMYB44* promoter upstream of the translation start codon was inserted between *Hin*dIII and *Bam*HI sites of pBI101.2, and then transformed into *A. tumefaciens* GV3101.

The complete vectors were introduced into potato by *Agrobacterium*-mediated transformation as previously described (Chronis *et al.*, 2013).

### Protein structure analysis and phylogenetic tree analysis

Predicted StMYB44 and homologs from Arabidopsis, tomato, tobacco, and cotton were aligned by using Clustal Omega (http://www.ebi.ac.uk/Tools/msa/clustalo/). The phylogenetic tree was built with the Molecular Evolutionary Genetics Analysis (MEGA) software. Bootstrap analysis of the phylogenetic tree was performed using 100 replicates.

### RNA extraction, library construction, RNA-Seq, and quantitative RT-PCR

Total RNA was extracted from roots of potato plants by using an E.Z.N.A.^®^ Total RNA Kit I (Omega Bio-tek, Norcross, GA, USA). A 5 μg aliquot of total RNA was used for library preparation as previously described (Zhong *et al.*, 2011). Sequencing was conducted on an Illumina HiSeq2500 at the Genomics Resources Core Facility of Weill Cornell Medical College.

Total RNA samples were treated with RQ1 DNase (Promega, Madison, WI, USA) for 30 min to remove genomic DNA, and then converted into cDNA using iScript™ Reverse Transcription Supermix (Bio-Rad, Hercules, CA, USA). Quantitative real-time PCR (qRT-PCR) was conducted in a CFX Connect Real-Time System with iTaq Universal SYBR Green Supermix (Bio-Rad). The thermal cycle involves 95 °C for 3 min, and 40 cycles of 95 °C 15 s and 60 °C for 60 s, followed by melt curve analysis to verify the specificity of amplification. The ΔΔCt method was used to calculate RT-PCR results with the potato *Actin* gene as an internal control.

### RNA-Seq data processing and analysis

Libraries were sequenced on a HiSeq2500 (Illumina) using 101 base, single-end sequencing, and the quality of RNA-Seq data was determined by using FASTQC (v 0.10.1) (http://www.bioinformatics.babraham.ac.uk/projects/fastqc/). Reads were mapped to the reference *S. tuberosum* Group Phureja DM1-3 genome assembly PGSC v4.03 pseudomolecules (http://solanaceae.plantbiology.msu.edu/pgsc_download.shtml) using TopHat2 (Kim *et al.*, 2013), allowing up to two mismatches. Differentially expressed genes were identified using cuffdiff following normalization of transcript count information to RPKM (reads per kilobase of exon model per million mapped reads) (Mortazavi *et al.*, 2008). Genes with a *P*-value <0.05 were considered to be differentially expressed.

### GUS staining

β-Glucuronidase (GUS) activity was assayed as previously described (Jefferson *et al.*, 1987) in transgenic potato seedlings, leaves, flowers, and tubers expressing the *Pro*_*MYB44*_*:GUS* chimeric gene using two independent transgenic lines for analysis.

To compare expression of the *StMYB44* promoter upon Pi starvation, transgenic seedlings were transferred onto fresh medium with Pi (Hoagland solution) or medium without Pi (Hoagland solution without Pi) and grown for 5 d. GUS activity in the seedlings was examined as above.

### Subcellular localization of StMYB44

*Agrobacterium* cells containing *35S:StMYB44-GFP* and *35S:GFP* plasmids, respectively, were infiltrated into 4-week-old *Nicotiana benthamiana* leaves. Three days after infiltration, the leaves were detached and green fluorescent protein (GFP) signals were examined under a Leica TCS-SP5 confocal microscope (Leica Microsystems Exton, PA, USA) with excitation wavelength at 488 nm and emission wavelength at 500–520 nm.

Six-day-old transgenic Arabidopsis seedlings expressing the *35S:StMYB44-GFP* and *35S:GFP* transgenes were used to study subcellular localization. Nuclei of root cells were stained with DAPI solution at 10 μg ml^–1^ (w/v) for 10 min, and then washed three times with water. Transgenic Arabidopsis seedlings expressing *35S:GFP* were used as the control. GFP and DAPI signals were examined using a Leica TCS-SP5 confocal microscope with excitation wavelengths 488 nm for GFP and 405 nm for DAPI (Zhou *et al.*, 2011).

### Pi content determination

Pi content was determined as previously described (Jain *et al.*, 2007) by grinding 6–20 mg of fresh shoot or root samples to a fine power in liquid nitrogen. The ground samples were suspended in 500 µl of 1% glacial acetic acid and immediately frozen in liquid nitrogen again and thawed. After centrifugation at 13 000 rpm for 1 min, 50 µl of supernatant were used in a phosphomolybdate colorimetric assay (Ames, 1966). To make Pi contents comparable, seedlings of wild-type and individual transgenic potato lines were grown in the same Magenta box containing 4.3 g l^–1^ Murashige and Skoog (MS) salt, 0.17 g l^–1^ NaH_2_PO_4_·H_2_O, 0.1 g l^–1^ inositol, 0.4 mg l^–1^ thiamine HCl, 30 g l^–1^ sucrose, and 1.8 g l^–1^ gelrite. Two weeks after subculture, shoot and root samples were collected for Pi content determination.

### Protein–protein interaction by BiFC

The coding sequences of *StMYB44*, *AtWRKY6*(At1g62300), and *StWRKY6* (NM_001318697, initially named *StWRKY31*, but it is more similar to *AtWRKY6*), a homolog of *WKRY6* from potato, without stop codons were amplified by PCR and cloned into the *Kpn*I and *Xma*I sites of the bimolecular fluorescence complementation (BiFC) vectors pSPYCE and pSPYNE, respectively (Waadt *et al.*, 2008). After confirmation by sequencing, the vectors were transferred into *A. tumefaciens* GV3101 and agroinfiltrated into 4-week-old *N. benthamiana* leaves. Three days after infiltration, the leaf discs were detached and examined by confocal microscopy for the yellow fluorescent protein (YFP) signal with excitation wavelength at 488 nm and emission filter at 520 nm.

## Results

### Identification of Pi starvation-responsive genes in potato roots by RNA-Seq

To investigate the regulatory mechanism of potato in response to Pi deficiency, differentially expressed genes in roots under Pi-sufficient (Hoagland solution with 0.5 mM KH_2_PO_4_) and Pi-deficient (Hoagland solution without Pi) conditions were examined by RNA-Seq. A previous study on rice subjected to Pi starvation elucidated a 2- to 3-fold change in Pi content in shoots and roots, but substantial numbers of differentially expressed genes were not observed until 3–7 d (Secco *et al.*, 2013). Therefore, to obtain a relatively comprehensive list of genes involved in the responses triggered by Pi deficiency, plant materials examined in this study were collected 5 d after Pi withdrawal.

**Fig. 1.**
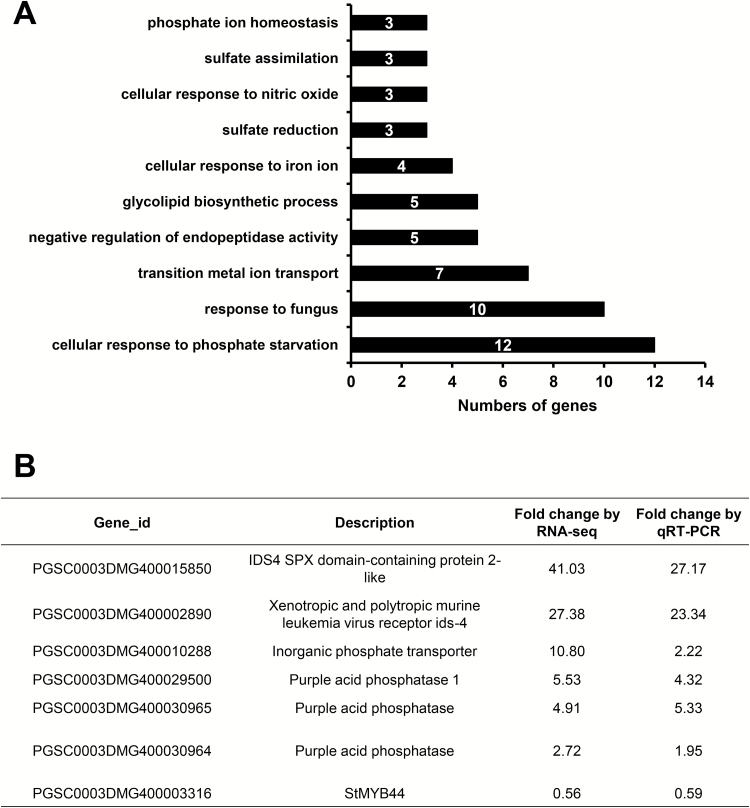
Identification of phosphate starvation-responsive genes in potato roots using RNA-Seq. (A) Functional categories of genes differentially expressed between Pi-sufficient and Pi-deficient potato roots. (B) Verification of gene expression by qRT-PCR. qRT-PCR was carried out with two biological repeats and three technical trials.

A total of 31.5 million reads were sequenced from six libraries generated from three biological repeats of Pi-deficient and sufficient samples. Statistical analysis indicated the differential expression of 359 genes upon Pi limitation, of which 221 genes were expressed at a minimum 1.6-fold higher level (see Supplementary Table S1 at *JXB* online). The rest of the genes were found to be reduced >1.6-fold upon Pi starvation as compared with control (Supplementary Table S2). Functional categorization of these genes revealed their involvement in diverse biological processes including cellular response to phosphate starvation and phosphate ion homeostasis (Fig. 1A). Further analysis indicated that several genes, including those encoding Inorganic Phosphate Transporter, four purple acid phosphatases, and three SPX domain-containing proteins were strongly up-regulated after Pi starvation treatment. In contrast, *PHOSPHATE2* (*PHO2*), a gene encoding a ubiquitin-conjugating E2 enzyme mediating the degradation of Phosphate Transporter 1 (PHT1) and PHOSPHATE1 (PHO1), was observed to be dramatically suppressed in potato roots (Supplementary Table S2). These results indicated that a 5 d Pi starvation treatment had successfully triggered comprehensive molecular responses in potato.

The replacement of phospholipids in membranes with glycolipids and sulfolipids is one of the typical responses of plants to Pi starvation (Härtel *et al.*, 2000). In this study, five genes, namely those encoding two glycosyltransferases, 1,2-diacylglycerol 3-beta-galactosyltransferase, digalactosyldiacylglycerol synthase 2 (DGD2), and riboflavin kinase/FMN adenylyltransferase, involved in the glycolipid biosynthetic process were identified. The Sulfate Transporter 3.4-encoding gene was observed to have greater abundance (17.5-fold increase) upon Pi starvation (Supplementary Table S1), suggesting an increase in S uptake or transport to meet the demand for the elevated biosynthesis of sulfolipids (Misson *et al.*, 2005).

Previous studies have indicated that starch accumulates in response to Pi deprivation (Calderon-Vazquez *et al.*, 2008; Hammond and White, 2008). The abundances of transcripts of starch synthase VI and two phosphofructokinase genes, involved in starch synthesis, were observed to be ~3-fold higher in Pi-depleted potato roots (Supplementary Table S1). The increased expression of these genes was also reported in Pi-deficient potato leaves (Hammond *et al.*, 2011).

The expression of several members of gene families involved in secondary metabolism and stress responses was altered by Pi starvation, including those encoding cytochrome P450s (eight genes), peroxidases (10 genes), and nodulins (five genes) (Supplementary Tables S1, S2), consistent with previous observations in maize, Arabidopsis, and rice (Misson *et al.*, 2005; Calderon-Vazquez *et al.*, 2008; Secco *et al.*, 2013).

### Verification of gene expression by quantitative RT-PCR

qRT-PCR was used to verify the expression of several genes potentially involved in Pi uptake and signaling, including those encoding IDS4 SPX Domain-containing Protein 2-Like, Xenotropic and Polytropic Murine Leukemia Virus Receptor IDS-4, Inorganic Phosphate Transporter, Purple Acid Phosphatase 1, and two purple acid phosphatases. Altered expression of these selected genes was consistent with that from the RNA-Seq approach although the scale of the fold changes differed between two approaches (Fig. 1B).

Among the Pi starvation-responsive genes, a number of targets, including seven up-regulated and nine down-regulated transcription factors, with potential signaling functions in response to Pi starvation were identified (Supplementary Table S3). *StMYB44* (PGSC0003DMG400003316), a potato homolog to *AtMYB44* and a member of the important MYB family subgroup 22, was down-regulated in roots by Pi starvation, as shown by both RNA-Seq and qRT-PCR (Fig. 1B). This gene was selected for a more comprehensive analysis of its involvement in regulation of Pi starvation responses.

### Isolation and structure analysis of potato StMYB44

**Fig. 2.**
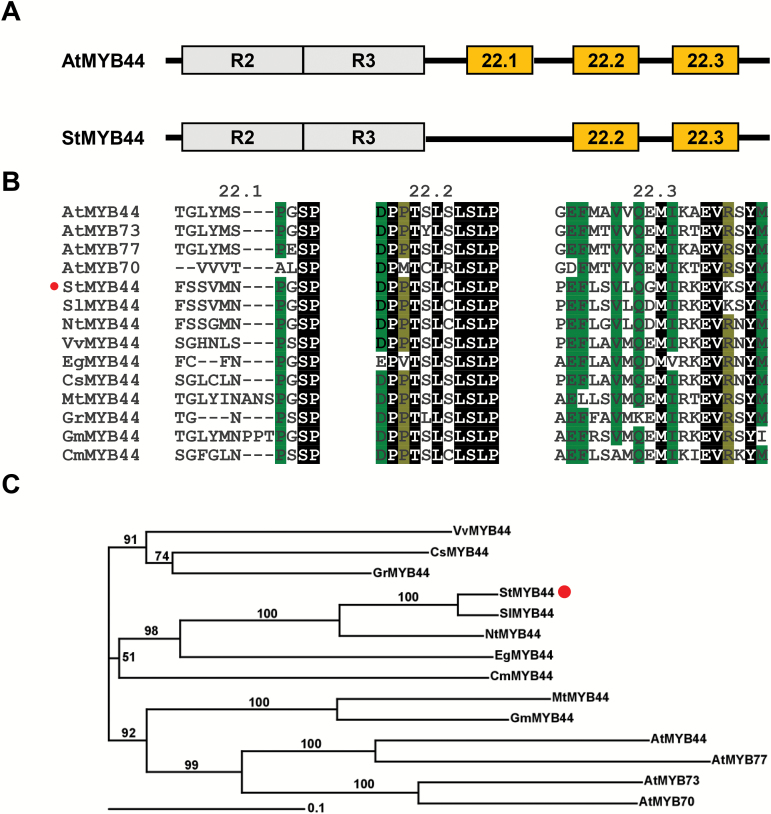
Isolation and analysis of StMYB44. (A) Schematic structures of StMYB44 and AtMYB44. (B) Conserved domains in MYB44 proteins. Alignment was conducted using Clustal Omega (http://www.ebi.ac.uk/Tools/msa/clustalo/). (C) Phylogenetic tree analysis of StMYB44 and homologs from other plant species. The GenBank accession numbers for the amino acid sequences are XP_006367421 for *Solanum tuberosum* MYB44, XP_004238123 for *Solanum lycopersicum* MYB44, NP_001311792 for *Nicotiana tabacum* MYB44, AT5G67300 for *Arabidopsis thaliana* MYB44, AT4G37260 for *Arabidopsis thaliana* MYB73, AT3G50060 for *Arabidopsis thaliana* MYB77, AT2G23290 for *Arabidopsis thaliana* MYB70, XP_002285015 for *Vitis vinifera* MYB44, XP_012851720 for *Erythranth eguttata* MYB44, NP_001275798 for *Citrus sinensis* MYB44, XP_003611666 for *Medicago truncatula* MYB44, XP_012451049 for *Gossypium raimondii* MYB44, NP_001238087 for *Glycine max* MYB44 (previously named MYB50), and NP_001315374 for *Cucumis melo* MYB44.

The ORF of *StMYB44* was isolated from potato cultivar Désirée by PCR. Sequencing analysis showed that the 963 bp long ORF encoded a protein of 320 amino acid residues with a predicted molecular mass of 35.02 kDa and an isoelectric point of 9.24 by using Compute pI/Mw software online (http://web.expasy.org/compute_pi/). The deduced protein shared 49% sequence identity with MYB44 in Arabidopsis. AtMYB44 belongs to R2R3-MYB subgroup 22 carrying R2 and R3 MYB repeat domains (Fig. 2A; Supplementary Fig. S1). Most members of this group in Arabidopsis contained the conserved motifs 22.1 (TGLYMSPxSP) and 22.3 (GxFMxVVQEMIxxEVRSYM) (Stracke *et al.*, 2001). Further analysis indicated that another conserved motif, 22.2, (D/EPP/MTxLSLP) is present between motifs 22.1 and 22.3 among the members of this group in Arabidopsis. StMYB44 carried the 22.2 and 22.3 motifs but lacked the 22.1 motif (Fig. 2A, B), indicating that it could have different physiological roles from its homologs in Arabidopsis. In addition, phylogenetic analysis showed that StMYB44 and its orthologs from tomato and tobacco form one clade with high bootstrap numbers (Fig. 2C), indicating that the divergence of StMYB44 occurred after the split of *Solanaceae* and *Brassicaceae*.

### Expression and subcellular localization of StMYB44

**Fig. 3.**
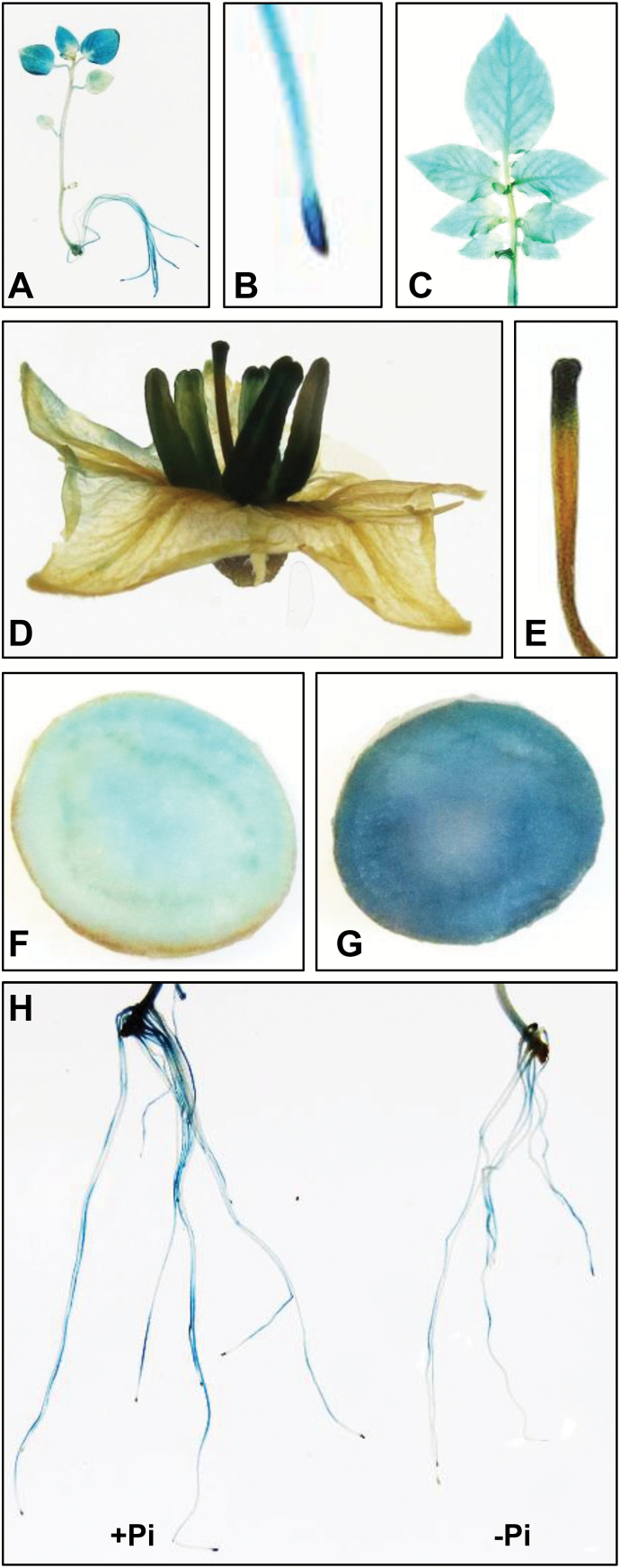
Tissue-specific expression pattern of *StMYB44* in Désirée. GUS staining of transgenic potato carrying the *Pro*_*StMYB44*_*:GUS* transgene. Tissues or organs at different stages included 2-week-old seedling (A), root tip from young seedling (B), mature leaf (C), flower (D), pistil (E), tuber stained for 3 h (F), and tuber stained for 6 h (G). (H) GUS activity in *Pro*_*StMYB44*_*:GUS* transgenic seedlings grown under Pi-sufficient and Pi deficient conditions.

To examine the tissue-specific expression patterns of *StMYB44*, a 1.5 kb fragment upstream of the start codon was fused to the GUS reporter gene, and transformed into potato. GUS assay showed *StMYB44* expression in almost all potato tissues, including young seedlings, roots, mature leaves, flowers, and tubers, although the expression in young leaves, root tips, stigma, and anthers was stronger than that in other tissues (Fig. 3A–G). Examination of GUS activity in *Pro*_*StMYB44*_*:GUS* seedlings grown in either Pi-sufficient or deficient conditions showed reduced staining in roots responding to Pi deficiency, further confirming the results of RNA-Seq and qRT-PCR (Fig. 3H).

**Fig. 4.**
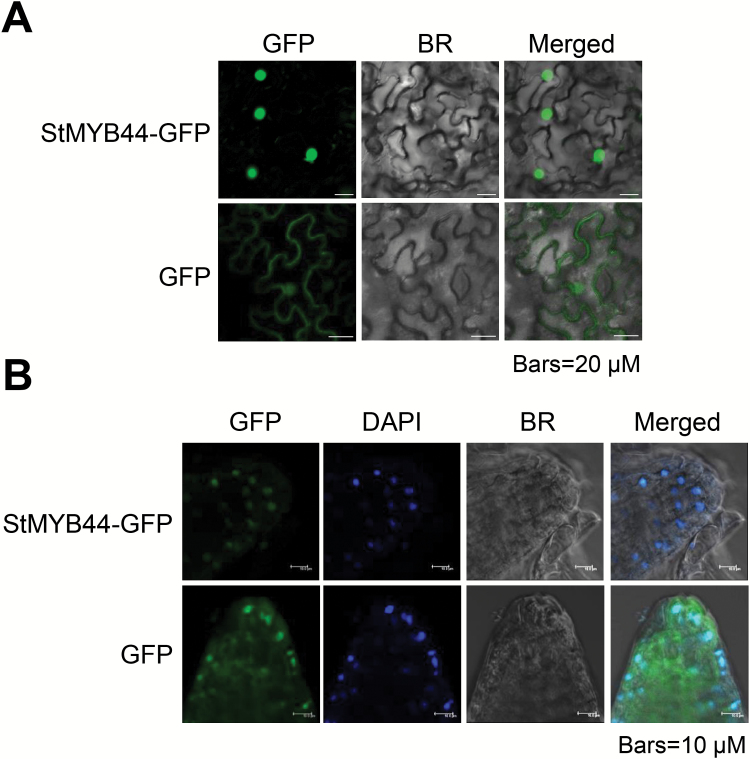
Subcellular localization of StMYB44. (A) Subcellular localization of StMYB44 in tobacco leaves. *Agrobacterium* carrying the *35S:StMYB44:GFP* and *35S:GFP* genes was infiltrated into *N. benthamiana* leaves. Images were taken 2 d after agroinfiltration by confocal microscopy. Scale bars=20 µm. (B) Subcellular localization of StMYB44 in Arabidopsis roots. Roots from 6-day-old transgenic Arabidopsis expressing *35S:StMYB44:GFP* and *35S:GFP* were stained with DAPI for 10 min. GFP and DAPI fluorescence signals were observed by confocal microscopy. Scale bars=10 µm.

To determine the subcellular localization of StMYB44, a StMYB44:GFP fusion protein was expressed in tobacco leaves by agroinfiltration. Confocal microscopic analysis showed the exclusive accumulation of StMYB44:GFP in the nucleus, whereas only GFP protein driven by the same CaMV *35S* promoter was found in the cytosol and nucleus (Fig. 4A).

In addition, roots of 6-day-old seedlings of two stable transgenic Arabidopsis lines expressing *35S:StMYB44:GFP* were stained with DAPI, a reagent specifically staining the nucleus. The overlap of the GFP and DAPI signals verified the nuclear localization of the StMYB44 protein, consistent with its function and the transient localization studied in tobacco leaf. As a control, GFP was detected in both the cytosol and nucleus (Fig. 4B).

### Overexpression of StMYB44 results in low Pi accumulation in potato shoots

**Fig. 5.**
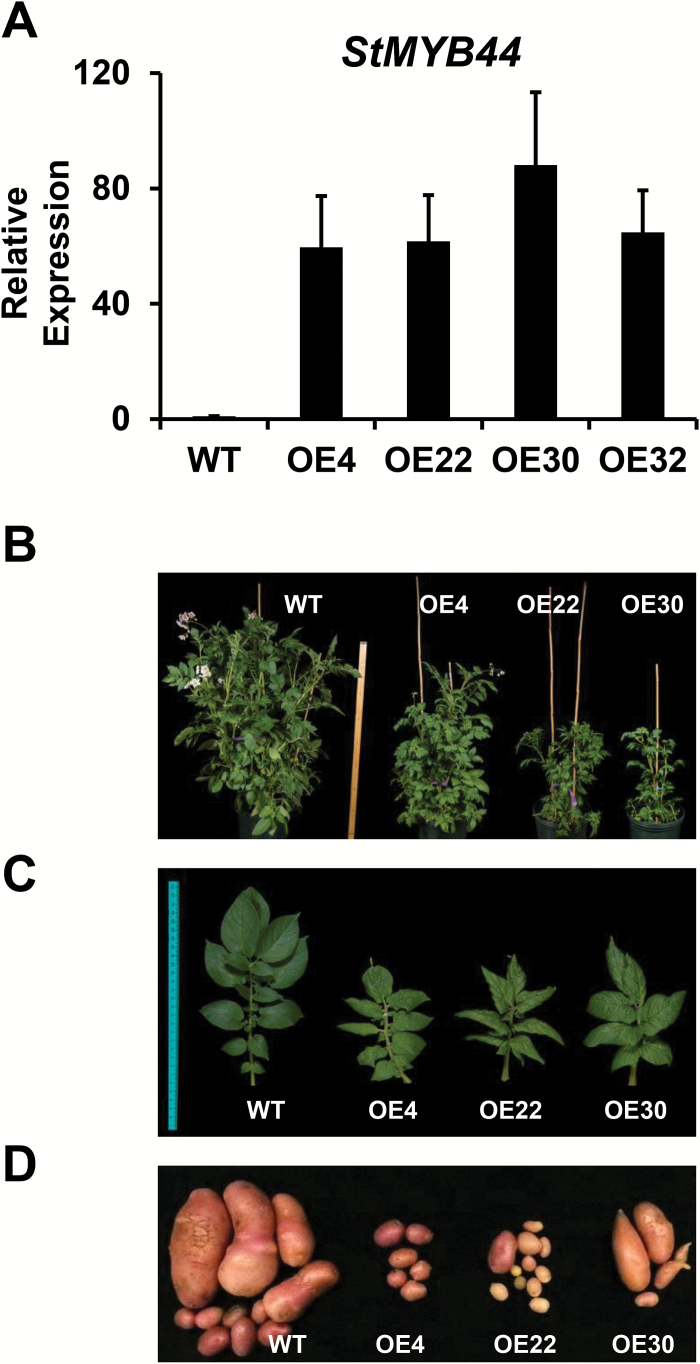
Overexpression of *StMYB44* in Désirée. (A) Expression of *StMYB44* in the independent transgenic potato plants by qRT-PCR analysis. *Actin* (XM_006350963) was used as an internal control to normalize the expression of the transgene. (B) Images of transgenic potato grown in soil. Leaves (C) and tubers (D) of transgenic and wild-type (WT) potato.

In order to reveal the physiological function of StMYB44, four transgenic potato lines with the highest expression level among 35 independent transgenic lines overexpressing *StMYB44:GFP* driven by a double 35S promoter were analyzed further (Fig. 5A). Under regular growth conditions in a greenhouse, the *StMYB44* overexpression lines were dwarf with small and curly leaves, and produced smaller and fewer tubers, indicating that elevated expression of *StMYB44* affected potato development and tuber yield (Fig. 5B–D).

**Fig. 6.**
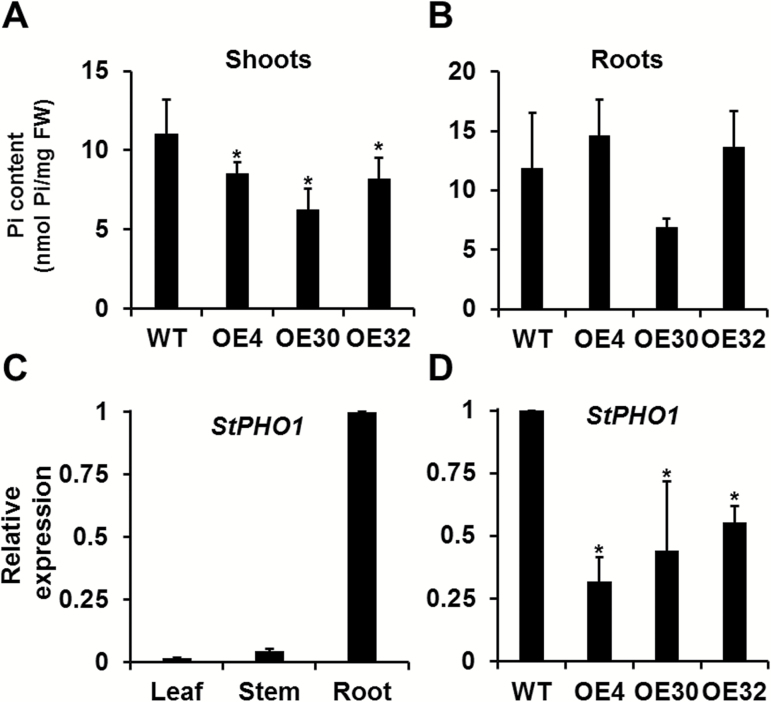
*StMYB44* overexpression leads to reduced Pi levels in shoots and down-regulation of *StPHO1* in roots. (A) Pi levels in transgenic potato shoots. (B) Pi content in transgenic roots. (C) Tissue-specific expression of *StPHO1*. (D) Expression of *StPHO1* in transgenic potato roots. Shoots and roots of wild-type (WT) and individual transgenic potato seedlings were collected for Pi content determination and gene expression analysis. The data represent means from three biological replicates. Error bars=SD. Student’s *t*-test was used to examine statistical significance. * indicates *P*<0.05.

Pi levels were measured in 14-day-old wild-type and transgenic potato seedlings grown on Pi-sufficient medium (MS medium containing 1.25 mM Pi KH_2_PO_4_). The shoot Pi contents of transgenic plants ranged from 6.25 nmol mg^–1^ FW to 8.54 nmol mg^–1^ FW and that of the wild type was 11.02 nmol mg^–1^ FW (Fig. 6A), while no significant difference was detected between the roots of wild-type and transgenic potato plants (Fig. 6B), indicating the negative effect of *StMYB44:GFP* overexpression on translocation of Pi from roots to shoots.

In order to dissect the downstream genes controlled by StMYB44, we compared global gene expression profiles between two independent transgenic lines (OE22 and OE30) and wild-type seedlings by RNA-Seq analysis. A total of 80.2 million reads were obtained from nine libraries generated from three biological repeats of wild-type and two transgenic lines. Of the 174 differentially expressed genes, 52 and 122 were discovered to be up-regulated and down-regulated, respectively, over 4-fold (Tables 1, 2). The RNA-Seq analysis showed that the averaged expression of *StMYB44* in the two chosen transgenic lines was increased by 44.3-fold, which is consistent with the qRT-PCR analysis of *StMYB44-*overexpressing lines, demonstrating the authenticity of RNA-Seq in identification of the differentially expressed genes in this study. Among the up-regulated genes in the *StMYB44-*overexpressing lines, *Purple Acid Phosphatase 3* (*PAP3*; PGSC0003DMG403007838), a gene involved in the release of the phosphate from phosphate ester under phosphate starvation conditions (Bozzo *et al.*, 2002; Y. Zhang *et al.*, 2014), was identified. At this point, it remained unclear whether the enhanced expression of *PAP3* was caused directly by the overexpression of *StMYB44* or a feedback response due to reduced Pi accumulation in shoots. More interestingly, the transcript abundance of potato *PHOSPHATE1* (*StPHO1*; PGSC0003DMG400017163) was observed to be reduced in the transgenic potato. In Arabidopsis, *PHO1* is responsible for loading Pi into the xylem in roots and its translocation from root to shoot; accordingly, mutation of this gene results in reduced Pi accumulation in shoot tissues (Poirier *et al.*, 1991; Hamburger *et al.*, 2002).

**Table 1.** Up-regulated genes in StMYB44-overexpressing lines

Gene_id	Gene	Fold change OE22/WT	*P-*value	Fold change OE30/WT	*P*-value	Average fold change
PGSC0003DMG400005840	Calcineurin B	inf	0.00005	inf	0.00005	inf
PGSC0003DMG400031059	Conserved gene of unknown function	inf	0.00005	inf	0.00145	inf
PGSC0003DMG400025420	Transposase	inf	0.00005	inf	0.00005	inf
PGSC0003DMG400003316	Tuber-specific and sucrose- responsive element-binding factor (TSF transgene)	30.36	0.00005	64.74	0.00005	44.34
PGSC0003DMG400019773	Sesquiterpene synthase 2	35.14	0.0001	18.37	0.00005	25.40
PGSC0003DMG400013696	Cytochrome P450	22.84	0.00005	27.68	0.00005	25.14
PGSC0003DMG400016180	Flowering locus T	15.35	0.00265	16.33	0.0002	15.83
PGSC0003DMG400010050	Proline oxidase/dehydrogenase 1	8.21	0.00005	30.42	0.00005	15.80
PGSC0003DMG400003954	Conserved gene of unknown function	15.54	0.00265	15.96	0.00285	15.75
PGSC0003DMG400025628	Pyridoxal-dependent decarboxylase, C-terminal sheet domain-containing protein	11.64	0.00005	18.77	0.00005	14.78
PGSC0003DMG400007796	DNA-directed RNA polymerase II largest subunit	12.22	0.00005	17.12	0.00005	14.46
PGSC0003DMG400000957	ATP-binding protein	15.04	0.00005	13.70	0.00005	14.35
PGSC0003DMG400014086	Gene of unknown function	22.40	0.0001	8.84	0.0009	14.07
PGSC0003DMG400024452	Pyridoxal-dependent decarboxylase, C-terminal sheet domain-containing protein	11.26	0.00005	17.14	0.00005	13.89
PGSC0003DMG400023230	2-Isopropylmalate synthase A	9.54	0.00005	15.91	0.00005	12.32
PGSC0003DMG400000776	Extensin (ext)	14.14	0.00005	8.73	0.00005	11.11
PGSC0003DMG400002046	Aspartic proteinase nepenthesin-1	9.29	0.00685	13.09	0.0005	11.03
PGSC0003DMG400005670	MAEWEST protein	8.62	0.00075	13.18	0.00385	10.66
PGSC0003DMG400024113	Gene of unknown function	11.46	0.0007	8.85	0.0008	10.07
PGSC0003DMG400024602	Conserved gene of unknown function	8.44	0.0051	11.99	0.0052	10.06
PGSC0003DMG400019274	Indole-3-acetic acid-amido synthetase GH3.6	8.17	0.00005	8.93	0.00005	8.54
PGSC0003DMG400031850	2-Hydroxyisoflavanone dehydratase	6.69	0.00345	10.20	0.0068	8.26
PGSC0003DMG400031437	Neryl diphosphate synthase 1	5.78	0.00005	9.51	0.00005	7.42
PGSC0003DMG400028593	Histidine-containing phosphotransfer protein	5.08	0.0048	9.91	0.00075	7.10
PGSC0003DMG400006319	Beta-glucosidase 01	4.51	0.00005	10.93	0.00005	7.02
PGSC0003DMG400015173	ATP-binding protein	10.03	0.00005	4.87	0.00005	6.99
PGSC0003DMG400015005	Heavy metal-associated domain- containing protein	4.37	0.0074	11.11	0.0005	6.97
PGSC0003DMG402002024	Zinc finger protein	5.75	0.00005	8.29	0.00005	6.91
PGSC0003DMG400006448	Caffeoyl-CoA *O*-methyltransferase	4.32	0.00005	10.85	0.00005	6.84
PGSC0003DMG400016722	Glutathione *S*-transferase	4.21	0.00875	10.95	0.00005	6.79
PGSC0003DMG400018579	Histidine phosphotransfer protein	5.52	0.00005	8.22	0.00005	6.74
PGSC0003DMG400019293	NAC domain-containing protein	7.66	0.00145	5.65	0.00095	6.58
PGSC0003DMG400023112	Kinesin	5.49	0.0009	6.93	0.0004	6.17
PGSC0003DMG400010713	Salt-responsive protein 2	8.54	0.00005	4.27	0.00005	6.04
PGSC0003DMG400002899	AP2/ERF domain-containing transcription factor	7.65	0.0001	4.76	0.0019	6.03
PGSC0003DMG400000493	Carbonic anhydrase	6.90	0.00005	5.22	0.00005	6.00
PGSC0003DMG400020156	Pectase lyase	5.96	0.00005	5.89	0.00005	5.92
PGSC0003DMG400011226	Sodium/potassium/calcium exchanger 6	4.66	0.00005	7.41	0.00005	5.87
PGSC0003DMG403007838	Purple acid phosphatase 3	4.37	0.00055	7.87	0.00005	5.87
PGSC0003DMG400003084	Two-component response regulator ARR8	7.72	0.00005	4.29	0.00005	5.76
PGSC0003DMG400024593	Glycosyltransferase UGT90A7	6.44	0.00005	5.04	0.00005	5.70
PGSC0003DMG400000730	Transcription factor	4.66	0.00005	6.56	0.00005	5.53
PGSC0003DMG400009268	Proteinase inhibitor	4.07	0.00005	7.15	0.00005	5.39
PGSC0003DMG400017189	Desacetoxyvindoline 4-hydroxylase	5.23	0.00035	5.11	0.00005	5.17
PGSC0003DMG400028229	Calcium-dependent protein kinase CDPK12	4.85	0.00005	5.12	0.00005	4.98
PGSC0003DMG400002520	Zinc finger protein	4.06	0.00005	5.48	0.00005	4.72
PGSC0003DMG400012977	VQ motif-containing protein	4.80	0.0067	4.61	0.0053	4.70
PGSC0003DMG400002519	Zinc finger protein	4.34	0.00005	4.88	0.00005	4.61
PGSC0003DMG400027212	ATP:citrate lyase	4.22	0.00015	4.86	0.00005	4.53
PGSC0003DMG400032780	Conserved gene of unknown function	4.68	0.00005	4.22	0.00005	4.44
PGSC0003DMG400026023	Nuc-1 negative regulatory protein preg	4.11	0.00105	4.73	0.0001	4.41
PGSC0003DMG400025479	PHAP2A protein	4.02	0.00005	4.51	0.00005	4.26

**Table 2.** Down-regulated genes in StMYB44-overexpressing lines

Gene_id	Gene	Fold change OE22/WT	*P*-value	Fold change OE30/WT	*P*-value	Average fold change
PGSC0003DMG400000207	Arabinogalactan peptide 16	0.00	0.0001	0.00	0.00005	0.00
PGSC0003DMG400019040	Gene of unknown function	0.00	0.0001	0.00	0.00005	0.00
PGSC0003DMG400020686	Gene of unknown function	0.00	0.00005	0.00	0.00005	0.00
PGSC0003DMG400014767	CND41, chloroplast nucleoid DNA- binding protein	0.02	0.00265	0.02	0.00265	0.02
PGSC0003DMG400011740	SGA rhamnose:beta-solanine/beta- chaconine rhamnosyltransferase	0.02	0.00005	0.02	0.00005	0.02
PGSC0003DMG400004143	SF16 protein	0.03	0.00265	0.03	0.00005	0.03
PGSC0003DMG400011334	Phylloplanin	0.06	0.00005	0.02	0.00265	0.04
PGSC0003DMG400020677	Conserved gene of unknown function	0.05	0.0028	0.04	0.00265	0.04
PGSC0003DMG400024770	Conserved gene of unknown function	0.04	0.00005	0.04	0.00005	0.04
PGSC0003DMG400014104	Patatin-2-Kuras 4	0.05	0.00005	0.05	0.00005	0.05
PGSC0003DMG400023922	Cytoplasmic small heat shock protein class I	0.11	0.00005	0.03	0.00005	0.05
PGSC0003DMG400030957	Cysteine proteinase	0.05	0.00265	0.06	0.00015	0.05
PGSC0003DMG400000123	Calcium-transporting ATPase, endoplasmic reticulum-type	0.07	0.00005	0.06	0.00005	0.06
PGSC0003DMG400006782	Conserved gene of unknown function	0.09	0.00005	0.05	0.00005	0.07
PGSC0003DMG400000984	3-Oxo-5-alpha-steroid 4-dehydrogenase family protein	0.05	0.0028	0.10	0.0068	0.07
PGSC0003DMG400039214	Arachidonic acid-induced DEA1	0.04	0.00005	0.14	0.00005	0.08
PGSC0003DMG400010048	Conserved gene of unknown function	0.08	0.00005	0.08	0.00005	0.08
PGSC0003DMG400011749	UDP-galactose:solanidine galactosyltransferase	0.05	0.00005	0.14	0.00005	0.08
PGSC0003DMG402017090	Patatin-04/09	0.08	0.00005	0.08	0.00005	0.08
PGSC0003DMG400010067	DNA-binding protein	0.10	0.0004	0.07	0.00015	0.08
PGSC0003DMG400011750	Cytochrome P-450	0.07	0.00005	0.10	0.00005	0.08
PGSC0003DMG400016458	Multi-antimicrobial extrusion family protein	0.06	0.0002	0.12	0.00005	0.09
PGSC0003DMG400011752	Cellulose synthase	0.06	0.00005	0.12	0.00005	0.09
PGSC0003DMG400029503	ETAG-A3	0.09	0.00005	0.08	0.00005	0.09
PGSC0003DMG400026404	Fragment	0.09	0.00005	0.08	0.00005	0.09
PGSC0003DMG400000048	Cysteine synthase	0.08	0.00005	0.10	0.00005	0.09
PGSC0003DMG400024983	Tuber-specific and sucrose- responsive element-binding factor	0.12	0.00005	0.08	0.00005	0.10
PGSC0003DMG402008890	Aldo-keto reductase family 4 member C10	0.13	0.00005	0.07	0.00005	0.10
PGSC0003DMG400011751	2-Oxoglutarate-dependent dioxygenase	0.09	0.00005	0.10	0.00005	0.10
PGSC0003DMG400004616	Invertase inhibitor	0.12	0.0077	0.08	0.00315	0.10
PGSC0003DMG400009033	Myb 12 transcription factor	0.07	0.00005	0.13	0.00005	0.10
PGSC0003DMG400002495	C2H2L domain class transcription factor	0.11	0.00005	0.09	0.00005	0.10
PGSC0003DMG400021142	DWARF1/DIMINUTO	0.10	0.00005	0.11	0.00005	0.10
PGSC0003DMG400022933	Auxin-induced beta-glucosidase	0.07	0.00005	0.14	0.00005	0.10
PGSC0003DMG400012797	Short-chain dehydrogenase/ reductase family protein	0.11	0.00005	0.10	0.00005	0.10
PGSC0003DMG400018930	Proteinase inhibitor I4, serpin	0.10	0.0053	0.11	0.0007	0.10
PGSC0003DMG400002028	Cytoplasmic small heat shock protein class I	0.15	0.00015	0.07	0.003	0.10
PGSC0003DMG400031792	Endo-1,4-beta-glucanase	0.11	0.00005	0.10	0.00005	0.11
PGSC0003DMG400003411	DNA-damage-inducible protein f	0.15	0.00005	0.08	0.00005	0.11
PGSC0003DMG400011350	OrfB protein	0.10	0.00005	0.11	0.00005	0.11
PGSC0003DMG400012763	C-4 sterol methyl oxidase	0.10	0.00005	0.12	0.00005	0.11
PGSC0003DMG400014339	Remorin	0.13	0.00005	0.09	0.00005	0.11
PGSC0003DMG400032817	Squamosa promoter binding	0.16	0.00005	0.07	0.00285	0.11
PGSC0003DMG400017505	Nam 11	0.13	0.00135	0.09	0.00435	0.11
PGSC0003DMG400012183	Endo-1,4-beta-glucanase	0.11	0.00605	0.12	0.0008	0.11
PGSC0003DMG401019681	Serine-threonine protein kinase, plant-type	0.10	0.00345	0.12	0.00005	0.11
PGSC0003DMG400010215	Cysteine protease	0.08	0.00005	0.15	0.00005	0.11
PGSC0003DMG400020777	Gene of unknown function	0.13	0.00005	0.10	0.00005	0.11
PGSC0003DMG400014347	PAR-1c protein	0.19	0.00005	0.07	0.00005	0.11
PGSC0003DMG400014543	Monoglyceride lipase	0.10	0.00005	0.13	0.00005	0.12
PGSC0003DMG400023419	Receptor kinase THESEUS 1	0.13	0.00015	0.11	0.0001	0.12
PGSC0003DMG400000523	Kinesin light chain	0.24	0.00005	0.06	0.0002	0.12
PGSC0003DMG400018140	Cytochrome P450 71A4	0.14	0.0084	0.11	0.0048	0.12
PGSC0003DMG400021814	Conserved gene of unknown function	0.18	0.0004	0.09	0.00395	0.12
PGSC0003DMG400007552	Conserved gene of unknown function	0.10	0.00005	0.15	0.00005	0.13
PGSC0003DMG400001544	Conserved gene of unknown function	0.12	0.0008	0.14	0.00005	0.13
PGSC0003DMG400024362	Anthranilate *N*-benzoyltransferase protein	0.24	0.00005	0.07	0.00005	0.13
PGSC0003DMG400015230	Pectate lyase	0.18	0.00005	0.09	0.00005	0.13
PGSC0003DMG401028252	Beta-fructofuranosidase	0.15	0.00005	0.11	0.00005	0.13
PGSC0003DMG400000719	Sec14 cytosolic factor	0.15	0.00005	0.12	0.00005	0.13
PGSC0003DMG400005526	Cytochrome P450	0.21	0.00005	0.08	0.00005	0.13
PGSC0003DMG400005734	FK506-binding protein	0.21	0.00005	0.08	0.00005	0.13
PGSC0003DMG400031763	Conserved gene of unknown function	0.12	0.00005	0.16	0.00005	0.14
PGSC0003DMG400028622	Acyl-protein thioesterase	0.10	0.00005	0.18	0.00005	0.14
PGSC0003DMG400019429	Conserved gene of unknown function	0.16	0.00005	0.12	0.0001	0.14
PGSC0003DMG400030784	Glutaredoxin family protein	0.17	0.0004	0.11	0.0007	0.14
PGSC0003DMG400012147	Conserved gene of unknown function	0.19	0.00015	0.10	0.00705	0.14
PGSC0003DMG400006221	Conserved gene of unknown function	0.15	0.00005	0.14	0.00005	0.14
PGSC0003DMG400001598	Snakin-2	0.14	0.00005	0.15	0.00005	0.15
PGSC0003DMG400027047	UPF0497 membrane protein	0.14	0.00005	0.15	0.00005	0.15
PGSC0003DMG402003937	P69E protein	0.18	0.00395	0.12	0.00735	0.15
PGSC0003DMG401031196	WRKY transcription factor 16	0.20	0.00005	0.11	0.00005	0.15
PGSC0003DMG400005633	Conserved gene of unknown function	0.22	0.00005	0.10	0.00005	0.15
PGSC0003DMG400007621	GAST1 protein	0.18	0.00045	0.12	0.0009	0.15
PGSC0003DMG400004493	GATA domain class transcription factor	0.24	0.00005	0.09	0.00055	0.15
PGSC0003DMG400027937	Conserved gene of unknown function	0.19	0.00105	0.12	0.0009	0.15
PGSC0003DMG400003848	Sugar transporter	0.19	0.00005	0.12	0.00005	0.15
PGSC0003DMG400004009	Phospholipase C	0.17	0.00005	0.14	0.00005	0.15
PGSC0003DMG400029937	ZIP family metal transporter	0.16	0.00005	0.14	0.00005	0.15
PGSC0003DMG402012192	Zinc finger protein	0.11	0.00005	0.23	0.00005	0.16
PGSC0003DMG400003626	Lactoylglutathione lyase	0.16	0.00005	0.16	0.00005	0.16
PGSC0003DMG400018565	Alcohol dehydrogenase	0.13	0.00005	0.21	0.00005	0.16
PGSC0003DMG400013828	Vacoular processing enzyme 1	0.21	0.00005	0.13	0.00005	0.16
PGSC0003DMG400025896	Proteinase inhibitor 1	0.18	0.00005	0.16	0.00005	0.16
PGSC0003DMG400002156	C-4 sterol methyl oxidase 2	0.23	0.00005	0.12	0.00005	0.17
PGSC0003DMG400018611	Glycosyl transferase family 17 protein	0.22	0.0001	0.13	0.00125	0.17
PGSC0003DMG402008895	Tropinone reductase 1	0.21	0.00005	0.14	0.00005	0.17
PGSC0003DMG400010223	Phytophthora-inhibited protease 1	0.24	0.00005	0.12	0.00005	0.17
PGSC0003DMG400020084	Zinc finger protein	0.24	0.00005	0.12	0.00015	0.17
PGSC0003DMG402000097	Conserved gene of unknown function	0.22	0.00205	0.14	0.00135	0.17
PGSC0003DMG400028295	Gene of unknown function	0.21	0.00005	0.14	0.00005	0.17
PGSC0003DMG400016977	Diphosphoinositol polyphosphate phosphohydrolase	0.23	0.0005	0.13	0.0013	0.17
PGSC0003DMG401019343	DNA-binding protein	0.18	0.00005	0.17	0.00005	0.18
PGSC0003DMG400014027	Germin	0.21	0.00005	0.14	0.00005	0.18
PGSC0003DMG400014173	Polyphosphoinositide-binding protein	0.18	0.00005	0.18	0.00005	0.18
PGSC0003DMG400017398	Snf1-kinase beta subunit, plants	0.18	0.00005	0.18	0.00005	0.18
PGSC0003DMG400014894	Membrane protein	0.20	0.0035	0.16	0.002	0.18
PGSC0003DMG400000715	Conserved gene of unknown function	0.18	0.0001	0.18	0.00005	0.18
PGSC0003DMG400017163	Xenotropic and polytropic murine leukemia virus receptor pho1	0.24	0.00005	0.14	0.00005	0.18
PGSC0003DMG400016651	Transcription factor RF2b	0.23	0.00005	0.15	0.00005	0.18
PGSC0003DMG400005035	ARF GAP-like zinc finger-containing protein ZIGA3	0.25	0.00005	0.14	0.00005	0.19
PGSC0003DMG400001529	Acidic 27 kDa endochitinase	0.23	0.00005	0.15	0.00005	0.19
PGSC0003DMG400021603	Hydroxyproline-rich glycoprotein (HRGP)EEYAN	0.16	0.00005	0.22	0.00005	0.19
PGSC0003DMG402027687	Wound-inducible carboxypeptidase	0.19	0.00005	0.19	0.00005	0.19
PGSC0003DMG400009892	Prolyl endopeptidase	0.22	0.00005	0.16	0.00005	0.19
PGSC0003DMG400015726	Glutathione *S*-transferase	0.17	0.0004	0.22	0.00005	0.19
PGSC0003DMG400030172	Aspartic proteinase oryzasin-1	0.20	0.00005	0.19	0.00005	0.20
PGSC0003DMG400029620	Chalcone synthase 1B	0.19	0.00005	0.21	0.00005	0.20
PGSC0003DMG400032182	Non-specific lipid-transfer protein	0.20	0.00465	0.20	0.0019	0.20
PGSC0003DMG400007018	3-Phosphoshikimate 1-carboxyvinyltransferase, chloroplastic	0.21	0.00005	0.19	0.00005	0.20
PGSC0003DMG400015169	Esterase	0.21	0.00005	0.20	0.00005	0.21
PGSC0003DMG400014093	Flavonol synthase	0.20	0.00005	0.22	0.00005	0.21
PGSC0003DMG400021423	Homeodomain leucine-zipper 1	0.23	0.00005	0.19	0.00005	0.21
PGSC0003DMG400005470	Rab GTPase activator	0.24	0.00005	0.20	0.00005	0.22
PGSC0003DMG400019110	Chalcone synthase 2	0.24	0.00005	0.20	0.00005	0.22
PGSC0003DMG400009959	Ornithine decarboxylase	0.22	0.00005	0.22	0.00005	0.22
PGSC0003DMG402024767	Pectinesterase	0.22	0.00005	0.23	0.00005	0.22
PGSC0003DMG400011502	PEP carboxylase kinase	0.23	0.00005	0.22	0.00005	0.23
PGSC0003DMG400010034	Photoreceptor-interacting protein	0.22	0.00005	0.25	0.00005	0.23
PGSC0003DMG400022459	BY-2 kinesin 5	0.22	0.00005	0.24	0.00005	0.23
PGSC0003DMG400006185	Skp1 1	0.25	0.00005	0.22	0.00005	0.23
PGSC0003DMG400020253	Ribonucleoside-diphosphate reductase small chain	0.22	0.00005	0.24	0.00005	0.23

Potato StPHO1, sharing 67% amino acid identity with the Arabidopsis PHO1 (Supplementary Fig. S2), is predominantly expressed in potato roots (Fig. 6C), similar to the expression pattern of *PHO1* in Arabidopsis (Hamburger *et al.*, 2002), and is expected to confer a similar Pi-translocating function in potato. qRT-PCR was used to confirm the decreased expression of *StPHO1* detected by RNA-Seq, and the result showed that the expression of *StPHO1* was significantly reduced in the *StMYB44*-overexpressing potato roots (Fig. 6D). Since *PHO1* is the only identified gene with a known function related to Pi loading and translocation, the lowered Pi accumulation in the transgenic potato shoots could be attributed to the reduction of *StPHO1* expression, caused by the overexpression of *StMYB44*, suggesting that StMYB44 negatively regulates Pi translocation from roots to shoots by specifically suppressing the expression of *StPHO1*.

### Knock-out of StMYB44 by using the CRISPR/Cas9 system

The CRISPR/Cas9 system was employed to generate *StMYB44* knock-out lines in potato (Xing *et al.*, 2014) using the nucleotide sequence from 376 to 396 of *StMYB44* mRNA as guide RNA. After *Agrobacterium*-mediated transformation, a total of 11 kanamycin-resistant potato lines were obtained, and PCR genotyping detected *Cas9* in all transgenic lines (Supplementary Fig. S3A). A fragment of ~300 bp of *StMYB44* harboring the target region was amplified by PCR and sequenced (Supplementary Fig. S3B), indicating that 9 out of 11 lines carried mutant *StMYB44* alleles, with a 81.8% frequency of gene editing for this CRISPR/Cas9 system in potato. The targeted mutations ranged from 2 to 120 deleted nucleotides in all of these nine transgenic plants. In addition to deletions, insertions of nucleotide A or T were observed in four lines, C12, C17, C19, and C21, consistent with reports on other plant species (H. Zhang *et al.*, 2014; Ma *et al.*, 2015). Désirée is a tetraploid potato cultivar, and single nucleotide polymorphism (SNP) analysis of the cloned *StMYB44* fragments indicated that it had eight alleles of *StMYB44* (Supplementary Fig. S4). The sequencing results also indicated that wild-type alleles of *StMYB44* were still present in C3, C12, C17, and C21, showing that not all the alleles in transgenic potato were modified. To better understand the degree of the reduced expression of *StMYB44* in these lines, the expression level of wild-type *StMYB44* in leaves was measured by RT-PCR. Expression of wild-type *StMYB44* was hardly detected in the selected transgenic plants, while it remained high in wild-type plants (Supplementary Fig. S3C).

All *StMYB44* knock-out lines displayed no visible phenotype compared with the wild type under normal conditions, except one line, C14, which grew more slowly and carried smaller leaves than the wild type. No statistically significant difference was observed in Pi content in shoots and roots between three selected *StMYB44* knock-out lines and wild-type plants (Supplementary Fig. S5A, B). *StPHO1* was expressed similarly in both the transgenic potato roots and wild-type roots (Supplementary Fig. S5C), implying that knock-out of *StMYB44* is not enough to increase transcription of *StPHO1*, probably due to the presence of other negative transcription factors. Expression of *StPHO1* and Pi contents were similar in C14 and the wild type (Supplementary Fig. S5), suggesting that the abnormal phenotypic change of C14 was most probably caused by an insertion in a development-related gene instead of Pi metabolism.

### StMYB44 interacts with AtWRKY6 and StWRKY6 in vivo

**Fig. 7.**
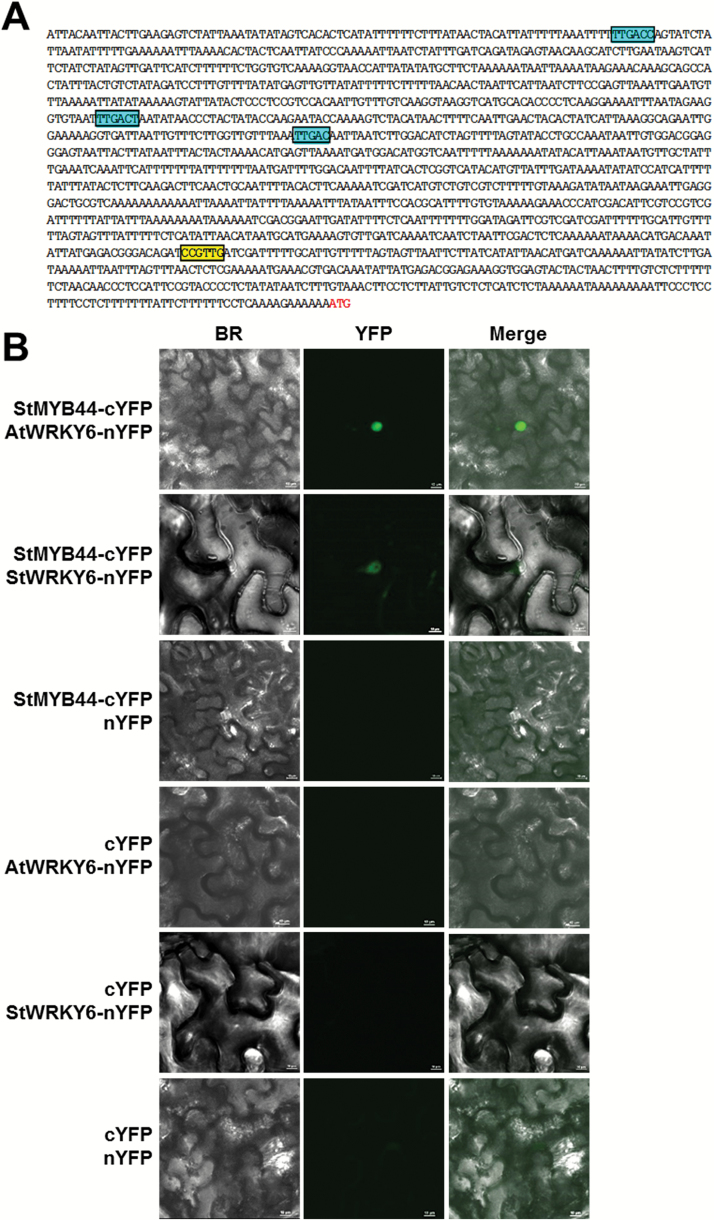
StMYB44 interacts with AtWRKY6 and StWRKY6 *in vivo*. (A) Analysis of *cis*-elements in the *StPHO1* promoter. Only the W-box (blue box) and MBSI (yellow box) are shown. (B) Interactions of StMYB44 with AtWRKY6 and StWRKY6. A suspension of *Agrobacterium* cells (OD_600_=0.05 for each strain) was infiltrated into *N. benthamiana* leaves. Infiltrated leaf discs were detached and examined 3 d after infiltration.

Transcription factor AtWRKY6 binds to the W-boxes in the *AtPHO1* promoter and suppresses its expression in Arabidopsis (Chen *et al.*, 2009). Three W-boxes [(T)TGAC(C/T)] were identified in the 1.5 kb *StPHO1* promoter. In addition, a MYB-binding site I (MBSI) (CCGTTG), located 297 bp upstream of ATG, was identified in the potato *PHO1* (Fig. 7A). EMSA showed that Arabidopsis AtMYB44 directly binds to the MBSI motif (Jung *et al.*, 2012). It is highly possible that StMYB44 could regulate the transcription of *StPHO1* by directly binding to the MBSI motif as StMYB44 shares a high amino acid identity with AtMYB44 in the DNA-binding domains (Supplementary Fig. S1). Furthermore, the juxtaposition of two kinds of *cis*-elements led to speculation that StMYB44 interacts with AtWRKY6 to form a protein complex. This hypothesis was tested by fusing StMYB44 to the C-terminal half of YFP (StMYB44–cYFP), and AtWRKY6 and StWRKY6 to the N-terminal half (AtWRKY6–nYFP and StWRKY6–nYFP), respectively, and introduced into tobacco leaves by agroinfiltration. Co-expression of 35S:StMYB44–cYFP and 35S:AtWRKY6–nYFP, 35S:StMYB44–cYFP, and 35S:StWRKY6–nYFP resulted in a YFP signal in the nucleus (Fig. 7B). In contrast, no YFP signal was detected when combinations of 35S:nYFP and 35S:cYFP, 35S:StMYB44–cYFP and 35S:nYFP, 35S:cYFP and 35S:AtWRKY6–nYFP, and 35S:cYFP and 35S:StWRKY6–nYFP were expressed (Fig. 7B). These results demonstrated that StMYB44 interacts with AtWRKY6 and StWRKY6 *in planta*.

## Discussion

The regulatory mechanism of Pi starvation responses in plants has been the subject of intensive investigation. A number of key genes controlling Pi homeostasis and regulation have been identified in plants, mainly in Arabidopsis and rice, where mutants or transgenic plants are easier to acquire (Rubio *et al.*, 2001; Hamburger *et al.*, 2002; Chen *et al.*, 2009; Rouached *et al.*, 2010; López-Arredondo *et al.*, 2014). However, relatively limited information is available in species in which either a mutant collection does not exist or the generation of transgenics is more difficult. Here, the RNA-Seq approach was selected which has been widely used to study dynamic changes in gene expression in Pi-deficient plants, including Arabidopsis, white lupin, rice, and wheat (Lan *et al.*, 2012; Oono *et al.*, 2013*a*, *b*; O’Rourke *et al.*, 2013; Secco *et al.*, 2013). In the present study, a transcriptomic analysis was conducted of potato root in response to low P_i_ by RNA-Seq, and *StMYB44*, one of the transcription factor genes identified, was characterized in more detail.

### Genetic responses to Pi starvation in potato roots

A total of 359 genes were identified to be P_i_ deficiency responsive (Supplementary Tables S1, S2). The number of differentially expressed genes is higher than that (147) in Pi-deficient maize roots at day 3 after treatment, and lower than that (967) in maize roots at day 6 after treatment, as reported in previous studies (Calderon-Vazquez *et al.*, 2008). These genes functioned in diverse biological processes as shown by Gene Ontology (GO) analysis, including Pi homeostasis and other related metabolic processes (Fig. 1A), suggesting that Pi deficiency causes profound changes in these processes in potato roots. Common genetic responses to Pi starvation in potato and other plant species regarding Pi uptake, distribution, and signaling, lipid metabolism, carbon assimilation, and other stress pathways were observed, supporting the notion that Pi-deficient responses are largely conserved among plants (Franco-Zorrilla *et al.*, 2004; Calderon-Vazquez *et al.*, 2008). Interestingly, differentially expressed genes involved in two biological processes, cellular responses to fungus and nitric oxide, and negative regulation of endopeptidase activity, were also identified. The connections between Pi starvation and these biological processes were not reported in previous studies and thus could be interesting to explore in future research.

Although the focus of this study was on molecular responses to Pi starvation in potato roots, a comparison of our results with one of the previous studies in which the potato leaf was analyzed led to the identification of similar or distinct metabolic pathways between the two tissues. A few pathways involved in starch accumulation, protein degradation, lipid metabolism, and S uptake were activated, and the expression of the associated genes encoding starch synthase, phosphofructokinase, E3 ubiquitin ligase, and ubiquitin-protein ligase, plus *SUT3*, was found to be increased in both leaf and root tissues (Supplementary Table S1) (Hammond *et al.*, 2011). In contrast, different responses to Pi deficiency were also observed between shoot and root in potato. For example, the patatin-encoding gene and four *Phospholipase A1* (*PLA1*) genes were down-regulated in roots, while two *Phospholipase D* (*PLD*) genes were up-regulated in potato leaves under Pi-limiting conditions (Hammond *et al.*, 2011). As the main tuber storage proteins, patatins also possess phospholipase A2 (PLA2) activity (Senda *et al.*, 1996). PLDs hydrolyze structural phospholipids, while PLAs hydrolyze galactolipids more efficiently than phospholipids and are involved in auxin signaling in roots (Rietz *et al.*, 2010; Canonne *et al.*, 2011). The up-regulation of *PLD* genes in shoot and down-regulation of *PLA* genes in root indicated that the breakdown of phospholipids mainly occurs in the shoot while an altered auxin signaling mediated by PLAs occurss in root during Pi deficiency.

### StMYB44 is a negative regulator of Pi transport from roots to shoots

The major purpose of this study was to identify the novel signaling transducers in potato in response to Pi deficiency. MYBs are among the well-characterized transcription factors regulating Pi deficiency responses. According to the phenotypic effect of either overexpression or knock-out of these *MYB* genes on Pi homeostasis, *PHR1/OsPHR2*, *PSR1*, *AtMYB2*, and *OsMYB2P-1* had positive effects on Pi uptake or transport (Wykoff *et al.*, 1999; Rubio *et al.*, 2001; Zhou *et al.*, 2008; Dai *et al.*, 2012; Baek *et al.*, 2013), whereas *MYB62* negatively regulates Pi content in the shoot by reducing Pi uptake and acid phosphatase activity (Devaiah *et al.*, 2009). Our study demonstrated that StMYB44 plays a negative role in Pi transport from root to shoot by regulating the transcription of *PHO1*. Genetic analysis has already demonstrated that the transcription of *PHO1* is negatively regulated by the transcription factor AtWRKY6 in Arabidopsis (Chen *et al.*, 2009). Regulation of *PHO1* by StMYB44, a transcription factor from a different family from AtWRKY6, in plant roots indicated an additional regulatory mechanism of Pi transport, expanding our knowledge of the physiological functions of this gene family.

 It is important to realize that the strong shoot morphological alterations in the *StMYB44* overexpression lines are less likely to be caused by the reduced allocation of Pi from root to shoot. How StMYB44 mediates the growth and development of potato is worth future exploration, although current interest is focused on its involvement in Pi metabolism.

### Control of PHO1 expression by multiple transcription factors

PHO1 is responsible for Pi transport from roots to shoots by loading Pi to the xylem (Hamburger *et al.*, 2002). Transcription of *PHO1* is under tight control in response to Pi availability since *PHO1* was induced by Pi starvation and quickly recovered by Pi resupply in rice (Secco *et al.*, 2013). A number of *cis*-elements, which can be recognized by several regulatory proteins including MYB transcription factors, in the promoter of Arabidopsis *PHO1* were predicted. Similarly, the promoter region of *StPHO1* was predicted to harbor several regulatory *cis*-elements, including binding sites for both WRKY transcription factors (W-box) and MYB transcription factors, suggesting that MYB transcription factors, such as StMYB44, could be involved in the regulation of *StPHO1* expression by binding directly to its *cis*-elements. Moreover, it is known that not only can WRKY transcription factors physically interact with other members in the same family but they can also interact with transcription factors or regulatory proteins in other families. For example, AtWRKY6 and AtWRKY42 interacted with each other in Arabidopsis (Chen *et al.*, 2009). HvWRKY38 interacted with Barley Prolamin-Box Binding Factor (BPBF), a non-WRKY transcription factor, to repress the expression of *Amy32b* in barley aleurone cells (Zou *et al.*, 2008). These results demonstrated that these interactions could play an important role in the regulation of genes controlled by WRKY proteins, as documented previously (Chi *et al.*, 2013).

This study showed that StMYB44 physically interacts with AtWRKY6 and StWRKY6 *in vivo*. To our knowledge, this is the first time that these two classes of transcription factors, WRKY and R2R3 MYB, were demonstrated to interact in the nucleus. These interactions allow us to propose that StMYB44 forms a complex with StWRKY6 in potato to regulate *StPHO1* expression synergistically. Under normal conditions, expression of *PHO1* is tightly controlled by the transcriptional complex to avoid overaccumulation of Pi in shoots, while upon Pi deficiency, removal of repressors StWRKY6 and/or StMYB44 leads to a lowered abundance of the transcription factor complex, facilitating the transcription of *PHO1* and associated Pi transport from root to shoot. Further studies, such as functional analysis of other Pi deficiency-responsive transcription factors or identification of StMYB44-interacting proteins, would not only advance our knowledge on the regulatory mechanism of potato in response to Pi starvation, but also shed light on the selection of candidate genes that could be used for genetic enhancement of Pi deficiency tolerance in potato and other crops.

RNA-Seq data in this study have been deposited in GenBank with accession no. SRP083083.

## Author contributions

XZ and CZ conceived the project and designed the experiments; XZ and MZ performed the experiments; JH and MI provided technical assistance; XZ, LL, and CZ analyzed the data and wrote the article.

## Supplementary Material

Supplementary Data
