# Supplementary material for: StMYB44 negatively regulates phosphate transport by suppressing expression of PHOSPHATE1 in potato
Source: J Exp Bot. 2017 Mar 1;68(5):1265–81. doi: 10.1093/jxb/erx026 (PMC5441854; doi:10.1093/jxb/erx026)
Supplement: Supplementary Data [file erx026_Supplementary_Data.zip › supplementary_figures_S1_S5.pdf]

```
                                                    R2
ATMYB44     --------MADRI KGPWSPEEDEQLRRLVVKYGPRNWTVISKSIPGRSGKSCRLRWCNQL  52
ATMYB70     -MSGSTRKEMDRI KGPWSPEEDDLLQSLVQKHGPRNWSLISKSIPGRSGKSCRLRWCNQL  59
ATMYB73     -MSNPTRKNMERI KGPWSPEEDDLLQRLVQKHGPRNWSLISKSIPGRSGKSCRLRWCNQL  59
ATMYB77     --------MADRV KGPWSQEEDEQLRRMVEKYGPRNWSAISKSIPGRSGKSCRLRWCNQL  52
STMYB44     MAAITQRKDSDRI KGPWSPEEDELLQTLVEKHGPRNWTLISKSVPGRSGKSCRLRWCNQL  60
                      :*: ***** ***: *: :* *:*****: ****:**************
                                          R3
ATMYB44     SPQVEH RPFSAEEDETIARAHAQFGNKWATIARLLNGRTDNAVKNHWNSTLKRK CGGYDH 112
ATMYB70     SPEVEH RGFTAEEDDTIILAHARFGNKWATIARLLNGRTDNAIKNHWNSTLKRK CSGGGG 119
ATMYB73     SPEVEH RAFSQEEDETIIRAHARFGNKWATISRLLNGRTDNAIKNHWNSTLKRK CSV--- 116
ATMYB77     SPEVEH RPFSPEEDETIVTARAQFGNKWATIARLLNGRTDNAVKNHWNSTLKRK CSGGVA 112
STMYB44     SPQVEH RAFTPEEDDTIIRAHAKYGNKWATIARLLSGRTDNAIKNHWNSTLKRK CPSMSE 120
            **:*** * *: ***:**  *:*::*******:***.******:*********** *

ATMYB44     RGYD--------------GSEDHRPVK---RSVSAGSPPVVTGLYMSPGSPTGSDVSDSS   155
ATMYB70     GGEEGQSCDFGGNGGYDGNLTDEKPLKRR----ASGGGGVVVVTAL---SPTGSDVSEQS   172
ATMYB73     ---EGQSCDFGGNGGYDGNLGEEQPLKRT----ASGGGGVSTGLYMSPGSPSGSDVSEQS   169
ATMYB77     VTTVTET-----------EEDQDRPKKRRSVSFDSAFAPVDTGLYMSPESPNGIDVSDSS   161
STMYB44     DLS---------------FETPQPPLKRS----SSVGPCTNFSSVMNPGSPSGSDLSDSS   161
                                . * *       :    .     :   **.* *:*:.*

ATMYB44     TIPI-----LPSVELFKPVPRPGAV---VLPLP----IETSSSSDDPPTSLSLSLPGADV   203
ATMYB70     QSSGSVLPVSSSCHVFKPTARAGGVVIE----S----SSPEEEEKDPMTCLRLSLPWVNE   224
ATMYB73     S---------GGAHVFKPTVRSEVT--------------ASSSGEDPPTYLSLSLPWTDE   206
ATMYB77     TIPS---PSSPVAQLFKPMPISGGFTVVPQPLP----VEMSSSSEDPPTSLSLSLPGAEN   214
STMYB44     LSG------FPQPLVYRPVPRTGGIFPLPPPPPPVKQIEIPSSVPDPPTSLCLSLPGSGS   215
                        :::*                       ..  ** * * ****

ATMYB44     SEESNRSHESTNINNTTS--SRHNHNNTV----SFMPFSGGFRGAIEEMGKSFPGNGGEF   257
ATMYB70     STT----------------------PPEL----FPVKREEEE-----KEREISGLGGDF   253
ATMYB73     TVRVNEP---VQLNQNTV--MDGGYTAEL----FPVRKEEQVEVEEEEAKGISGGFGGEF   257
ATMYB77     TS---------------S--SHNNNNNAL----MFPRFESQMKINVEERGGGGEGRRGEF   253
STMYB44     IEKPTQSPNSPPLPPPPLPVVDKPIPPSAAVMGHLPRSNQSYDFCAAPKSGEKQFFTPEF   275
                                                .                   :*

ATMYB44     MAVVQEMIKAEVRSYMTEMQRNNGGGFVGGFID---------NGMIP-MSQIGV-----G   302
ATMYB70     MTVVQEMIKTEVRSYMADLQLGNGGG---AGGGASSCMVQGTNGRNVGFREFI----GLG   306
ATMYB73     MTVVQEMIRTEVRSYMADLQRGNVGGSSSGGGGGGSCMPQSVNSRRVGFREFIVNQIGIG   317
ATMYB77     MTVVQEMIKAEVRSYMAEMQKTSGGFVVGGLYE---------SGGNGGFRDCGI---ITP   301
STMYB44     LSVLQGMIRKEVKSYMSGFEQN------------GICMQTD------AIRNAVIKRIGIS   317
            ::*:* **: **:***: ::                            : :

ATMYB44     RIE 305
ATMYB70     RIE 309
ATMYB73     KME 320
ATMYB77     KVE 304
STMYB44     KIE 320
            ::*
```

Supplemental Figure S1. Alignment of StMYB44 and members of subgroup 22 in *Arabidopsis*.

Protein sequences of StMYB44 (XP_006367421), MYB44 (AT5G67300), MYB70 (AT2G23290), MYB73 (AT4G37260), and MYB77 (AT3G50060) were aligned using Clustal Omega.

```
  PHO1    (1) MVKFSKELEAQLIPEWKEAFVNYCLLKKQIKKIKTSRKPKPASHYPIGHH
StPHO1    (1) MVKFSKELEAQLIPEWKDAFVNYWQLKKQVKKIKISKKPHHVHDGNSSLI

  PHO1   (51) SDFGRSLFDPVRKLARTFSDKLFSNSEKPEILQVRRRRGSSETGDDVDEI
StPHO1   (51) HDFGRSIFDSIRSFTITSNMKFHKSHEVSQVKSIIKEGENGEEQEEQEEI

  PHO1  (101) YQTE--LVQLFSEEDEVKVFFARLDEELNKVNQFHKPKETEFLERGEILK
StPHO1  (101) YETENELVQLFSEEDEVRLFFEMLDEELKKVNEFYKTKESEFLERGDILN

  PHO1  (149) KQLETLAELKQILSDRKKRNLSG-SNS---HRSFSSSVRNSDFSAGSPGE
StPHO1  (151) KQLQILLDLKQVLSDRRRKTLGSRSGSGFFSRSHSSSGRNSDFSETQSDC

  PHO1  (195) LSEIQSETS-RTDEIIEALERNGVSFIN-SATRSKTK-GGKPKMSLRVDI
StPHO1  (201) GSPTGTETISQTEEVIAALEKNGINFVNSASTRAKTKKGGKPKVAMRIDI

  PHO1  (242) PDAVAGAEGGIARSIATAMSVLWEELVNNPR----SDFTNWKNIQSAEKK
StPHO1  (251) PATTP------TRTIAAVTSMLWEDLVNNPKKDGPREYINKKKIQCAEKM

  PHO1  (288) IRSAFVELYRGLGLLKTYSSLNMIAFTKIMKKFDKVAGQNASSTYLKVVK
StPHO1  (295) IRGAFVELYRGLGLLKTYSSLNMVAFVKILKKFDKVAKQQSSANYLKQVK

  PHO1  (338) RSQFISSDKVVRLMDEVESIFTKHFANNDRKKAMKFLKPHQTKDSHMVTF
StPHO1  (345) RSHFISSDKVVRLMDEVESLFTQHFANSDRKKAMKFLRPQQNKESHMVTF

  PHO1  (388) FVGLFTGCFISLFVIYIILAHLSGIFTSSDQVSYLETVYPVFSVFALLSL
StPHO1  (395) FVGLFTGCFVTLFSVYAILAHLSGMFSTRTEAAYVETVYPVFSMFALLSL

  PHO1  (438) HMFMYGCNLYMWKNTRINYTFIFEFAPNTALRYRDAFLMGTTFMTSVVAA
StPHO1  (445) HLFMYGCNLFLWKGTRINYNFIFEFQPKTALKYRDAFLIGTCLMTSVVGA

  PHO1  (488) MVIHLILRASGFSASQVDTIPGILLLIFICVLICPFNTFYRPTRFCFIRI
StPHO1  (495) LVVHLILLSNGFSPSQVDAIPGILLLIFLALLICPLNVFYRPTRFYFLKV

  PHO1  (538) LRKIVCSPFYKVLMVDFFMGDQLTSQIPLLRHLETTGCYFLAQSFKTHEY
StPHO1  (545) IRNIVCSPFYKVLMVDFFMADQLTSQIPLMRHLESSACYFLAGSLTTHGL

  PHO1  (588) NTCKNGRYYREFAYLISFLPYFWRAMQCVRRWWDESNPDHLINMGKYVSA
StPHO1  (595) PTCKSGRMYRELAYVISFAPYYWRAMQCARRWFDESDINHLANLGKYVSA

  PHO1  (638) MVAAGVRITYARE-NNDLWLTMVLVSSVVATIYQLYWDFVKDWGLLNPKS
StPHO1  (645) MVAAGARLTYGREPDSQLWFSIVLVTSVIATVYQLYWDFVKDWGFFNLKS

  PHO1  (687) KNPWLRDNLVLRNKNFYYLSIALNLVLRVAWIETIMRFRVSPVQSHLLDF
StPHO1  (695) KNFLLRDELILKNKSIYYASIALNLVLRVAWVETVMHFNVGPFESHLLDF

  PHO1  (737) FLASLEVIRRGHWNFYRVENEHLNNVGQFRAVKTVPLPFLDRDSDG
StPHO1  (745) FLASLEVIRRGHWNFYRLENEHLNNVGKFRAVKTVPLPFRETDSDG
```

Supplemental Figure S2. Alignment of Arabidopsis PHO1 and StPHO1.

Predicted protein sequence of StPHO1 (PGSC0003DMG400017163) and *Arabidopsis* PHO1 were aligned by using Clustal Omega.

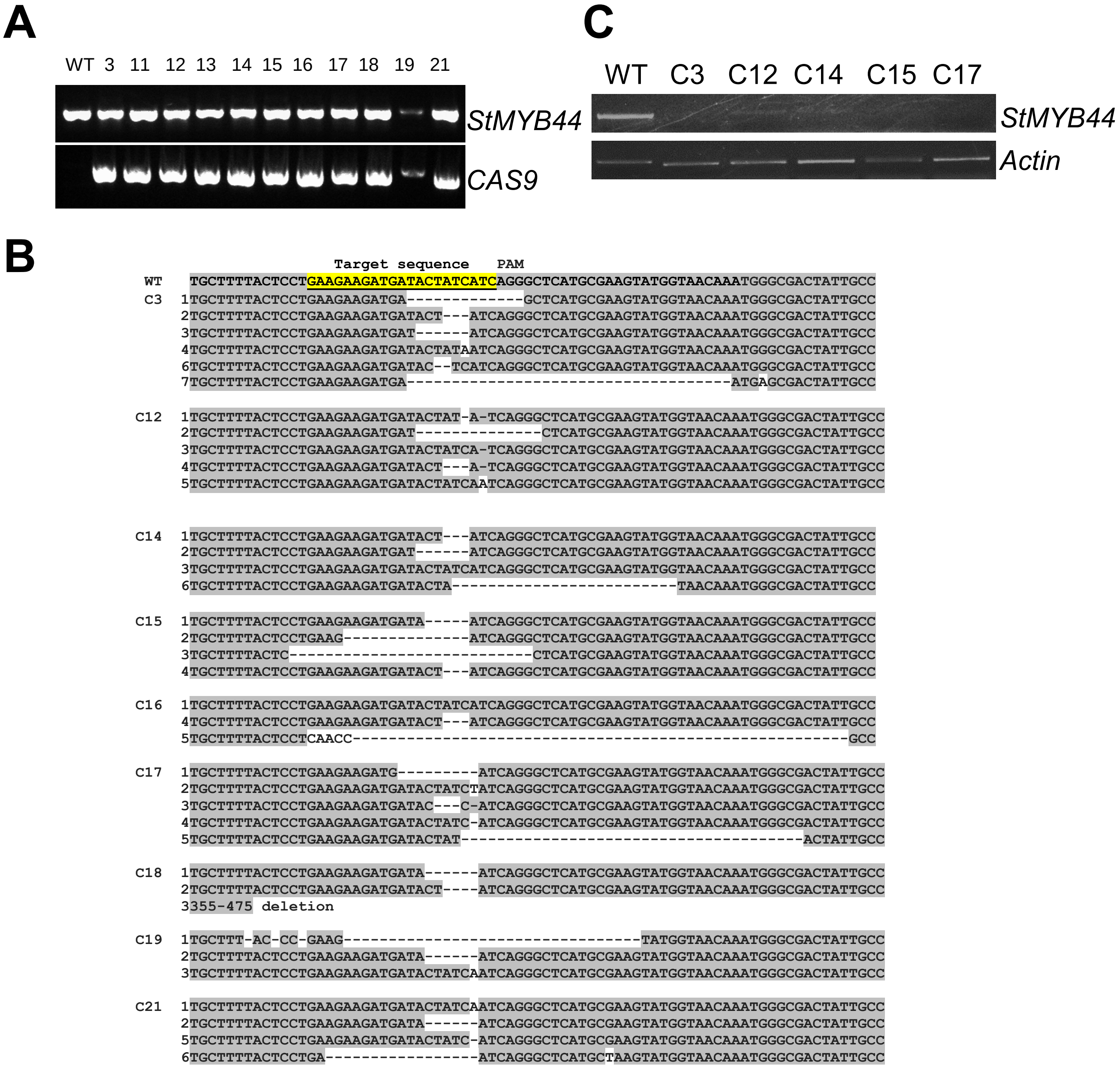

Supplemental Figure S3. Knock-out of *StMYB44* by CRISPR/Cas9 system

A. PCR genotyping of transgenic potato lines. Transgenic plants were identified by PCR with specific primers for *Cas9* gene. B. *StMYB44* sequences in transgenic potato lines. PCR fragments from WT and the individual transgenic lines were sequenced and aligned. The targeted sequence and PAM are underlined or indicated. C. Expression of *StMYB44* in transgenic lines by semi-quantitative RT-PCR.

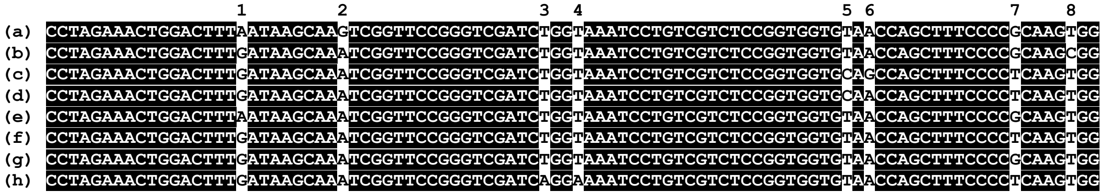

Supplemental Figure S4. Eight alleles of StMYB44 in Desiree based on SNPs**.**

Eight SNPs were identified by comparing the cloned *StMYB44* fragments (300bp) by using Clustal Omega.

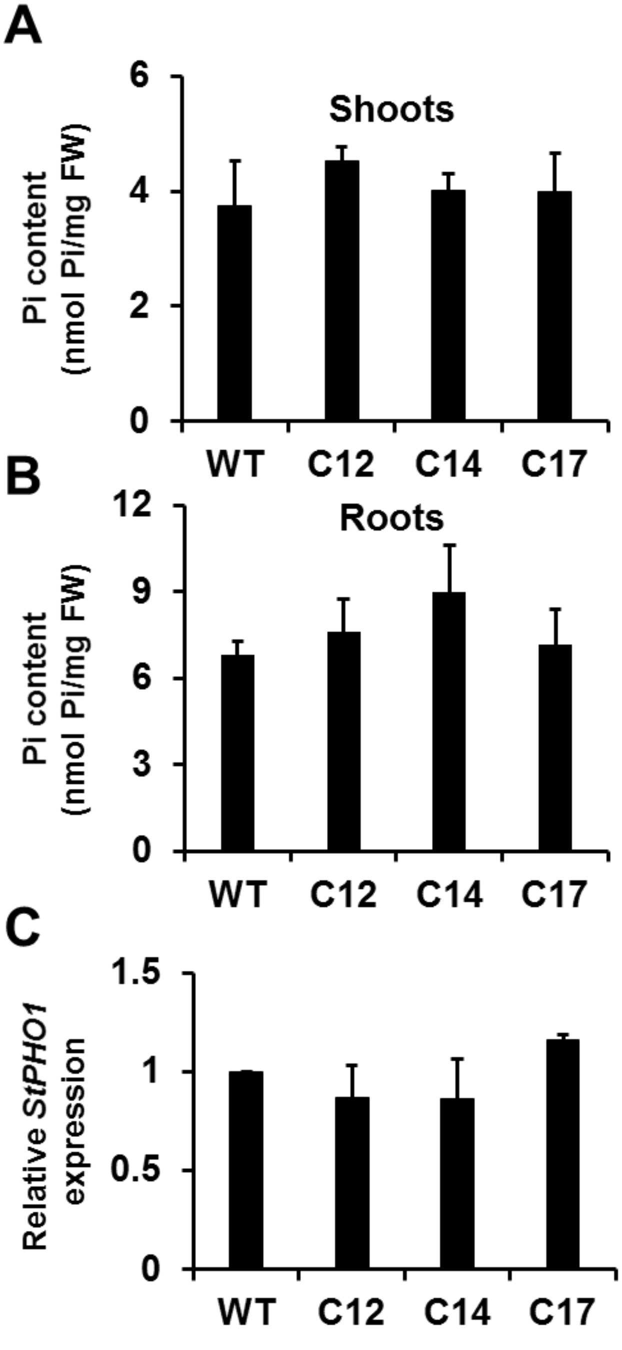

Supplemental Figure S5. Pi levels and expression of *StPHO1* remain unchanged in *StMYB44* knock-out lines

A. Pi content in transgenic shoots. B. Pi content in transgenic roots. C. Expression of *StPHO1* in transgenic potato roots by qRT-PCR. Pi content determination and gene expression analysis were conducted in shoots and roots of WT and individual transgenic potato seedlings grown on MS medium. The data represent means from three biological replicates.
